# Supplementary material for: Comparative performance of a commercial and in-house Mp1p antigen-detecting enzyme immunoassay for the rapid diagnosis of talaromycosis
Source: PLoS Negl Trop Dis. 2025 Aug 1;19(8):e0013248. doi: 10.1371/journal.pntd.0013248 (PMC12342302; doi:10.1371/journal.pntd.0013248)
Supplement: Attachment — Submitted filename: Mp1p EIA response to reviewers final.docx [file pntd.0013248.s001.docx]

**Response to Reviewers**

We are deeply thankful of the editors and reviewers for your comprehensive review and valuable input. Below are our point-by-point responses to your suggestions.

**Reviewer #1:**

**Methods**

Lines 137-139: Consider defining other specimens were considered clinical

- We have amended line 150-151 on page 8: “*The reference standard was isolation of T. marneffei in culture of blood or other clinical specimens* ***(including skin lesions, lymph nodes and bone marrow)…*”**.

Lines 139: Consider referencing another paper that describes clinical syndromes consistent with talaro for those readers who may not be as familiar with the disease

- We agree with your suggestion and have **cited on line 151, page 8** our recent review in *Clinical Infectious Diseases* which describes the clinical syndromes in different patient groups.

Lines 139-143: Consider describing a bit more about how the controls were randomly selected? Numbering system? Same with the cases.

- We have added to line 155-157, page 8: “***Cases and controls were randomly selected from batches of cases and controls based on availability of sample aliquot and volume without knowledge of clinical and laboratory characteristics of patients.*”.**

Lines 186-188: The authors may want to add a bit of reasoning into why likelihood ratios were chosen over the more commonly used predictive values. And a bit more commentary on how to use likelihood ratios.

- We used likelihood ratios rather than predictive values to describe test performance as likelihood ratios are independent of disease prevalence. They are more appropriate ways to interpret test performance generated from a retrospective case-cohort study design, where the **disease prevalence is artificially generated. Predictive values** are however prevalence-dependent and should only be used to interpret assay performance in a prospective assay validation study. We have added to line 206-209, page 10: “*In a retrospective case-cohort study - where the* ***disease prevalence is artificially generated****,* ***likelihood ratios*** *are* ***more appropriate ways to interpret assay performance than*** ***predictive values*** *which are prevalence-dependent and could be misleading*”.

**Results**

Reviewer #1: Lines 194-195: Consider removing this sentence and just citing figure after the next sentence for flow of manuscript. Consider doing the same for subsequent sections in the results sections.

- We appreciate this suggestion but prefer to cite in this style for clarity, and to draw the reader’s attention to the figures/tables.

**Conclusions**

Reviewer #1: Lines 270-271: Consider adding reference to end of sentence.

- We have now provided reference on line 295, page 16.

Lines 342-343: Consider removing the designation as an NTD since technically not considered as an NTD by WHO; although, it very likely should be.

- Although not yet recognised by the WHO, talaromycosis has been designated a neglected tropical disease by PLOS NTD journal, and the National Institute for Health (NIH) in the USA.
- We have added to the discussion on line 394-398, page 19: “*Finally,* ***we advocate*** *for the* ***WHO*** *to* ***recognize*** *talaromycosis as a* ***neglected tropical disease****, as this could serve as a catalyst for action and drive progress in research and development for talaromycosis*.”

**Editorial and Data Presentation Modifications**

Reviewer #1: Figure 1: Consider adding a bit more detail about randomization process.

- We have added to the figure legend of Figure 1 “*Cases and controls were randomly selected based on availability of sample aliquot and volume without knowledge of clinical and laboratory characteristics of patients*” ”.

Figure 4: Will ultimately defer to authors, but the value added from figure 4 may be minimal and could be removed or in supplemental material.

- We thank you for this suggestion, although we feel strongly that a Venn diagram is very useful to demonstrate the overlap or concurrence between two assays. We are happy to move it to supplemental material if strongly desired by the Editors.

**Summary and General Comments**

Reviewer #1:

- Lines 87-88: While vast majority of talaro occurs in those immunocompromised, disease in immunocompetent hosts have been documented. Consider modifying the end of the sentence to reflect this.

- We have amended line 91, page 6 to “*Talaromyces marneffei, formerly Penicillium marneffei, is a thermally dimorphic fungus which causes the invasive fungal disease talaromycosis* ***primarily*** *affecting the immunocompromised host*.”

- Lines 93-96: Since this is PLoS NTDs, maybe consider including a sentence after WHO FPPL about how it is absent on WHOs NTD list for advocacy purposes? Will leave up to authors, but just a thought if authors think it will add to their report.

- Thank you for this important point. As described above, we have added to the discussion on 394-398, page 19: “*Finally, we advocate for the WHO to recognize talaromycosis as a neglected tropical disease, as this could serve as a catalyst for action and drive progress in research and development for talaromycosis*.”

Lines 97-106: May want to include a bit of commentary about other promising point of care tests that the authors have worked on, but not commercially available yet.

- We have included a discussion of other emerging point-of-care tests in the discussion, on lines 379-390, page 19.

**Reviewer #2:**

The original manuscript by Barwatt and colleagues, which presents a comparative analysis of the diagnostic performance between a commercial Wantai EIA and an in-house EIA based on the Mp1p antigen of Talaromyces marneffei, is scientifically written, clear, concise, and focused. However, there remain several aspects that warrant further consideration, as outlined below:

1. Clarification of the Study’s Primary Objective:
It is important for the authors to more clearly articulate the main objective of this experiment. Given that both ELISA systems were developed based primarily on rabbit polyclonal antibodies against Mp1p, the resulting outcomes should reasonably align (as the authors have demonstrated), and significant discrepancies would not be expected. Therefore, the rationale and specific aims of the study should be explicitly stated.

- Thank you for this important point. We have added to lines 125-132: *“While both EIAs use* ***similar*** *antibodies, there are some* ***key differences*** *in the* ***protocols****. The Wantai Mp1p EIA* ***preloads*** *and* ***fixes polyclonal antibodies*** *(PAbs) onto the plate using* ***proprietary stabilized reagents****, allowing for* ***shelf-life stability*** *of up to* ***12 months****. In contrast, our in-house Mp1p EIA plates must be* ***preloaded overnight*** *or within a few days prior to use. The Wantai Mp1p EIA incorporates a* ***co-incubation step****, in which circulating* ***Mp1p antigens*** *in human samples* ***interact simultaneously*** *with the* ***anti-Mp1p detection antibody****. This approach* ***reduces*** *the total assay time from* ***6 to 2 hours*** *by eliminating the sequential incubation steps required in our in-house Mp1p EIA.”*

2. Experimental Methodology:
There is some ambiguity regarding the experimental procedures. The methods used for the Wantai EIA and the in-house Mp1p EIA appear to differ slightly in their details. To enhance clarity and accessibility for the reader, the authors are encouraged to provide a schematic diagram or flow chart that visually summarizes the experimental workflow, rather than relying solely on textual descriptions.

- We have now highlighted on lines 125-132 the differences in the protocols of the Wantai and our in-house Mp1p EIAs.

3. False Negative and False Positive Characteristics:
Under the section addressing the characteristics of false negatives and false positives, the manuscript would benefit from additional detail. The occurrence of false negatives is not entirely unexpected, given that the mechanisms underlying the secretion of mannoprotein antigens in the pathogenesis of T. marneffei are not yet fully understood. The authors may wish to interpret this within the framework of the damage-response model of T. marneffei pathogenesis. Furthermore, baseline antigen levels within populations residing in endemic areas remain to be thoroughly investigated.
In contrast, the issue of false positives requires a more comprehensive discussion. In particular, potential cross-reactivity involving the rabbit anti-Mp1p antibodies should be considered and addressed in greater depth.

- Thank you for this very important point. We have added a new paragraph to the discussion on lines 319-335, pages 16-17, describing the potential causes of the false negative and false positive cases in our study as follows: “*There are several possible causes for the false positives and false negatives observed in our study.* ***Heterophilic antibody interference*** *may have led to false positives due to non-specific binding of human antibodies with the rabbit-derived Mp1p antibodies. While previous studies do not report cross-reactivity of the Mp1p EIA with other major pathogenic fungi, including Cryptococcus, Candida, Histoplasma, and Aspergillus, there remains* ***the potential for cross-reactivity*** *with less common fungi (12). We* ***did not suspect low fungal burden*** *as a cause of false negatives in our study, as five of the six false negative cases were positive by blood culture. False negatives may have occurred in people with* ***prior exposure to T. marneffei****, due to* ***sequestration of Mp1p antigen to circulating antibodies****, forming immune complexes and reducing the levels of free Mp1p. Further investigation is needed to characterize the adaptative immune response to T. marneffei.* ***Variations*** *in the* ***Mp1p epitope*** *may have arisen as a result of* ***genetic diversity*** *across different* ***strains of T. marneffei****, potentially contributing to false negative results (21).”*
